# Pathological Role of Natural Killer Cells in Parkinson's Disease: A Systematic Review

**DOI:** 10.3389/fnagi.2022.890816

**Published:** 2022-05-18

**Authors:** Le Zhang, Yingshuang Zhang, Dongsheng Fan

**Affiliations:** ^1^Department of Neurology, Peking University Third Hospital, Beijing, China; ^2^Peking University Health Science Center, Beijing, China; ^3^Beijing Key Laboratory of Biomarker and Translational Research in Neurodegenerative Diseases, Beijing, China; ^4^Key Laboratory for Neuroscience, National Health Commission/Ministry of Education, Peking University, Beijing, China

**Keywords:** Parkinson’s disease, α-synuclein, natural killer cells, natural killer cell cytotoxicity, neuroinflammation

## Abstract

Parkinson's disease (PD) is one of the common neurodegenerative diseases that is characterized by selective degeneration of dopaminergic neurons in the substantia nigra, and misfolding of α-synuclein into aggregates is thought to contribute to its pathology. Studies have shown that immune-inflammatory responses are involved in the development of PD and play an important role in α-synuclein scavenge. Natural killer (NK) cells are first responders in immune cells and can directly promote immune defense mechanisms by cytotoxicity and by secreting cytokines. Recent discoveries suggest that NK cells are increasingly recognized in the pathological features of PD. However, the mechanisms underlying it have not been fully understood. In this review, we systematically retrieved and evaluated published evidence about the functions of NK cells in PD. We find alterations in the number of NK cells and cytotoxicity during the progression of PD, and it seems that NK cells play a neuroprotective role in PD pathogenesis, which may further reveal novel targets for the management and treatment of PD.

## Introduction

Parkinson's disease (PD) is one of the fastest-growing neurological diseases in terms of prevalence and mortality and is the second most common degenerative disease in the elderly with an increasing burden on healthcare systems (Poewe et al., 2017; Brakedal et al., 2021; Feigin et al., 2021). It is principally characterized by motor features, such as postural instability, bradykinesia, tremor, and rigidity. In addition, non-motor symptoms, such as hyposmia, anxiety, depression, and cognitive dysfunction, are also found to be increasingly prevalent over the course of PD development, which are gradually being paid more attention (Schapira et al., 2017). Evidence has demonstrated that progressive and selective loss of dopaminergic neurons in the substantia nigra pars compacta (SNpc) is the main cause of motor symptoms, but the clinical diagnosis of the pathological changes that had already occurred before the manifestation of PD poses a challenge to the early diagnosis and treatment of PD (Gruden et al., 2011; Poewe et al., 2017).

At present, it is generally believed that the abnormal accumulation of α-synuclein (α-syn) is one of the important etiological factors leading to the degeneration of dopaminergic neurons, which is closely related to the formation of the Lewy body (Vekrellis et al., 2011; Poewe et al., 2017). In addition, extensive neuroinflammation characterized by the innate and adaptive immune response is found to contribute to the extensive degeneration of dopaminergic neurons (Tan et al., 2020; Harms et al., 2021). Remarkably, the neurotoxicity of α-syn, neuroinflammation, and neurodegeneration can trigger a series of pathophysiological mechanisms both locally and systemically in PD (Wong and Krainc, 2017; Tan et al., 2020; Harms et al., 2021).

Recent advances in the field of neuroinflammation demonstrate that complex interactions between CNS-resident cells and peripheral immune cells are involved in the PD pathogenesis (Iba et al., 2020; Tan et al., 2020; Harms et al., 2021). However, the mechanism involved in the recruitment of peripheral cells into the CNS and whether it is a passive migration or active participation are still unclear.

Natural killer (NK) cells are members of the innate lymphoid cells (ILCs) family, and represent 5-20% of the peripheral lymphocytes in humans (Abel et al., 2018; Zitti and Bryceson, 2018). A variety of inhibitory or activating receptors are expressed on NK cells, which regulate cell functions by binding to specific ligands and play crucial roles in cellular signal transduction. When the cells downregulate the expression of major histocompatibility complex class I (MHC-I) molecules or upregulate the expression of NK cell-activating receptor ligands abnormally, they may become the immunological attack targets for NK cells. The mechanism of cytotoxicity of NK cells can be basically classified into three types: direct release of the lytic molecules such as perforin and granzyme, activation of the extrinsic apoptotic pathway through Fas ligand (FasL) and TNF-related apoptosis-inducing ligand (TRAIL), and antibody-dependent cell-mediated cytotoxicity (ADCC) effects (Abel et al., 2018; Prager and Watzl, 2019). In addition, NK cells can produce and respond to different cytokines and play a crucial role in immunomodulation (Zitti and Bryceson, 2018).

It has been reported that NK cells are capable of interacting with α-syn in the animal models of PD and modulating neuroinflammation in neurodegenerative diseases (Poli et al., 2013; Earls et al., 2020; Garofalo et al., 2020). The existence of a link between PD and NK cells had been originally suspected due to two main reasons One is that NK cell activity was found to be related to neurological diseases, particularly demyelinating diseases in the previous studies, and researchers speculated whether similar pathological changes or immune interactions might also be present in patients with PD (Bokor et al., 1993). On the other hand, epidemiological studies provided evidence for the fact that patients with PD were more likely to have a lower incidence of cancer, probably because increased activity of natural killer cells provides defense against the infiltration of tumor cells (Mihara et al., 2008). Based on these findings, several basic and clinical studies focused on the pathological role of NK cells and performed the beneficial exploration and the attempt in this context. However, research in this field is still in its infancy, and some of the findings might be controversial. Therefore, we conducted a systematic review of the relevant literature to investigate the alterations of NK cells in PD and discuss how NK cells can influence the disease onset and progression.

## Materials and Methods

This systematic review was reported according to the Preferred Reporting Items for Systematic Reviews and Meta-Analyses (PRISMA) guidelines.

### Literature Search

The search of PubMed, Embase, and Web of Science databases (from inception up to January 2022) was performed by two authors (ZL and ZYS) independently. The search strategy involved selecting the most relevant Medical Subject Headings (MeSH) and title/abstract text keywords used in combination or alone: “Parkinson's disease,” “NK cell,” “natural killer cell,” “alpha-synucleinopathy,” and “dopaminergic nerve cell” (detailed search strategy is described in Supplementary Table 1).

### Selection Criteria

To prove the eligibility of the studies, the following inclusion and exclusion criteria were formulated:

Inclusion criteria were as follows: (1) articles published in English, (2) original articles regarding animals and humans (peripheral lymphocyte analysis) and *in vitro* studies (cell cultivation) of natural killer cells where relevant, and (3) the research theme focused on “Parkinson's disease.” Exclusion criteria were as follows: (1) case report, case series, letter, poster, commentary, proceedings, laboratory science studies, review, and systematic review, (2) repeatedly published literature, unpublished data, or findings reported in abstract form only, and (3) literature of poor quality.

### Data Extraction

Data extraction from eligible articles was performed by two investigators (ZL and ZYS) independently. The following clinical information was extracted from the study: publication, region of the population, animal models of PD, number of patients and healthy controls, gender statistics, age at diagnosis, disease duration, disease severity, and the main results.

### Quality Assessment and Risk of Bias

Quality assessment was carried out by two authors (ZL and ZYS) independently. Modified Collaborative Approach to Meta-Analysis and Review of Animal Data from Experimental Studies (CAMARADES) and Newcastle-Ottawa Scale (NOS) were used as quality assessment tools for the animal experimental studies and clinical studies separately. Ten items are included in the modified CAMARADES tool: sample size calculation, random allocation to treatment or control, allocation concealment, blinded assessment of outcome, appropriate animal model, use of anesthetic without significant intrinsic neuroprotective activity, statement of control of temperature, compliance with animal welfare regulations, peer-reviewed publication, and statement of potential conflict of interests. Seven items are included in the NOS tool: adequate case definition, representativeness of the cases, selection of controls, definition of controls, comparability of cases and controls on the basis of the design or analysis (study controls for age or any additional factors), ascertainment of exposure, and same method of ascertainment for cases and controls and non-response rate. The total scores of the two scales are 10 and 9, respectively. The final scores of each study are described in Supplementary Tables 2, 3. Any discrepancies were resolved through discussion. If an agreement could not be reached, the disagreements were resolved with the help of a third author.

## Results

**Figure 1:**
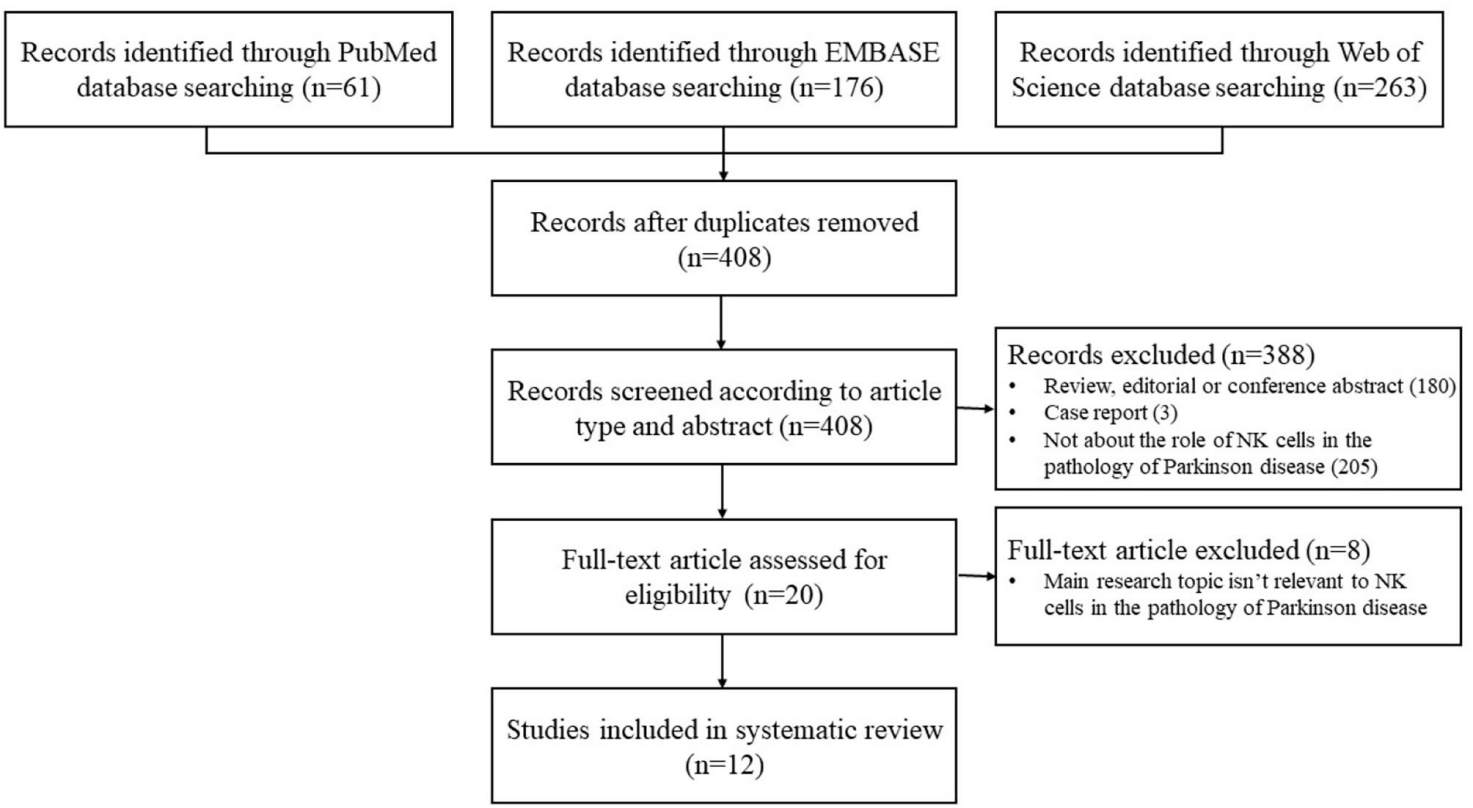
Flowchart of the study selection.

A total of 408 records were generated from the database search after removing the duplicates. After screening for title and abstract, 388 records were excluded. Full-text of the remaining 20 articles was assessed, and finally, a total of 12 studies were eligible for the systematic review after full-text screening (Figure 1).

### Animal Studies

We identified three studies on animals according to the inclusion criteria, and the main findings are summarized in Table 1. Two studies used a preformed fibril (PFF) α-syn injection model in C57BL/6J mice and transgenic mice that overexpressed human A53T α-syn mutant protein separately, and one study used a 6-hydroxydopamine (6-OHDA) injection model in Wistar rats. These two models are commonly used in the study of PD. For the PFF α-syn model, PFF α-syn acts as a template and induces the endogenous α-syn to accumulate into misfolded phosphorylated pathological aggregates in the substantia nigra (Earls et al., 2020). In the 6-OHDA model, 6-OHDA (a neurotoxin) is absorbed by dopaminergic neurons rich in monoamine oxidase in the substantia after injection, which can further convert into free radicals and cause damage to neurons; 6-OHDA does not induce the formation of α-syn aggregates or Lewy-like inclusions in PD (Grembecka et al., 2021).

**Table 1:** Main findings of animal studies.

**No**	**Author**	**Year**	**Animals**	**Models**	**Main results**	**PMID**
1	Earls	2019	C57BL/6J mice	PFF α-syn injection model	• Relative percentage of NK cells in PFF α-syn mice higher than monomer controls at 5 months; • Decreased numbers of NK cells in the blood of PFF α-syn mice compared with monomer controls; • No major changes in cytokines or chemokines in the serum of PFF α-syn mice compared with the monomer controls	31796095
2	Earls	2020	Transgenic mice (M83) overexpressing human A53T α-syn mutant protein	PFF α-syn injection model	Using the PFF α-Syn Mouse Model • NK Cells are found in the brains of the model; • NK cell depletion increased hind limb clasping and exacerbated motor deficits and motor function in the mice; • NK cell-deficient mice displayed significantly increased p-α-syn inclusions within the striatum, SNpc and brainstem; • Using the human NK Cell • NK92 cells efficiently internalized various sizes of α-syn (monomers, oligomers, and higher molecular weight fibrils) in a dose-dependent manner; • NK cells internalized α-syn in the cytoplasmic compartment and α-syn colocalized with both Rab7 and LC3B (the endosome and lysosome pathway marker); • Only α-syn aggregates significantly attenuated NK cell cytotoxicity in a dose-dependent manner; • Extracellular α-syn aggregates significantly decreased IFN-γ secretion in NK 92 cells; • The levels of NKG2A, and NKG2D receptors on NK cells were not altered by extracellular α-syn aggregate treatments;	31900358
3	Grembecka	2021	Wistar rats	6-OHDA injection model	• The NKCC was higher in 6-OHDA groups (dopamine depleted groups) in comparison to controls using a 51Cr release assay; • It seems that percentage of NK(CD3- CD161a+) cells in PBMC between 6-OHDA and control groups didn't show a significant difference	32648088

The first study (Earls et al., 2019) showed that intrastriatal injection of PFF α-syn could induce Lewy body–like pathology, and the percentage of NK cells in the mononuclear immune cells at 5 months post injection (p.i.) was relatively increased in the CNS parenchyma, which was confirmed by the flow cytometry analysis. In addition, characterization of the leukocytes in the peripheral blood showed that the number of NK cells decreased, but the percentage of the NK cell population did not show significant changes.

In the second study, Earls et al. confirmed the presence of NK cells in the SNs of both postmortem PD patients and M83 Tg mice based on immunohistochemistry (IHC) markers (Earls et al., 2020). More importantly, obvious clinical motor deficits and an increased number of p-α-syn inclusions within the striatum, substantia nigra pars compacta (SNpc), and brainstem were observed in PFF α-syn–injected M83 Tg mice after systemic NK cell depletion, which indicated the protective role of NK cells in PD. Moreover, researchers carried out cell-based assays and found the bidirectional effects between extracellular α-syn and NK cells: NK92 cells and primary human NK cells could internalize the extracellular α-syn and scavenge it through the endosome and lysosome pathway, while extracellular α-syn aggregates attenuated NK cell cytotoxicity (NKCC) and IFN-γ secretion without causing any significant changes in NKG2A and NKG2D receptors on the cell surface. It is an interesting phenomenon that NK cells are not aberrantly activated.

For the third study (Grembecka et al., 2021), we extracted information regarding the comparison between the PD model and the blank controls. It seems that the number of NK (CD3-CD161a+) cells in PBMC did not show any significant difference between the 6-OHDA groups and the controls (without *p*-values recorded). Furthermore, they observed elevated peripheral NKCC levels after 6-OHDA microinjection into the SNpc, which means NK cells might be actively involved in the progression of PD.

### Clinical Studies

Nine clinical studies explored the relationship between NK cells and sporadic Parkinson's disease according to the inclusion criteria, and the basic information and important findings of these studies are summarized in Tables 2, 3. Most of the included studies provided basic information regarding the subject number, gender, age at visit/onset of patients, disease duration, and H&Y/UPDRS-III (part III of the Unified Parkinson's Disease Rating Scale) assessment. NK cells are mainly characterized based on the cell number, cell cytotoxicity, and changes in the cell surface receptors.

**Table 2:** Basic information of clinical studies.

**No**	**Author**	**Year**	**Subjects**	**Gender (M/F)**	**Age (means±SD)**	**Disease duration (means±SD)**	**Disease severity (means±SD)**	**PMID**
1	Mihara	2008	20 patients 20 HC	PD: 9/11 HC: 11/9	PD: 70.7 ± 7.8 HC: 67.6 ± 9.4	5.4 ± 3.4	H&Y score: 2.9 ± 0.8	17702627
2	Niwa	2012	29 patients 30 HC	PD: 17/12 HC: 16/14	PD: 70.4 (mean) HC: 68.9 (mean)	6.38 ± 4.07	H&Y score: 2.63 ± 0.92 UPDRS III score: 23.92 ± 15.81	21929737
3	Stevens	2012	88 patients 77 HC	PD: 56/32 HC: 39/38	PD: 69 ± 9 HC: 67 ± 10	6 ± 5	H&Y score: 2 ± 0.7 UPDRS III score: 21 ± 10	22910543
4	Cen	2017	268 patients 268 HC	PD: 156/112 HC: 168/100	PD: 60.59 ± 11.11 HC: 59.41 ± 11.11	EOPD: 3.94 ± 2.32 LOPD: 4.95 ± 4.10	H&Y score: EOPD: 1.47 ± 0.51 LOPD: 1.93 ± 0.88 UPDRS III score: EOPD: 11.00 ± 4.63 LOPD: 12.89 ± 7.88	28791571
5	Sun	2019	127 patients 148 HC	PD: 75/52 HC: 84/64	PD: 62.7 ± 12.5 HC: 60.3 ± 13.4	4.4 ± 4.1	UPDRS III score: 17.9 ± 9.2	31930038
6	Anderson, K. M.	2020	1,314 patients 1,978 HC	-	-	-	-	32709660
7	Huang	2021	205 patients 233 HC	-	-	-	-	34483877
8	Konstantin Nissen	2022	78 patients 28 HC	PD: 39/39 HC: 15/13	age at visit: PD: 66.6 ± 9.5 HC: 64.2 ± 7.4 age at onset: PD: 60.4 ± 9.2	4.5 (IQR: 3-9)	H&Y score: 2 (median) UPDRS III score: 27.84 ± 14.29 MoCA: 26 (median)	35026420
9	Tian	2022	22 patients 18 HC	PD: 12/10 HC: 13/5	EOPD: 40.30 LOPD: 64.23 YHC: 40.30 EHC: 68.25	EOPD: 2.44 LOPD: 1.8	H&Y score: EOPD: 1.31 LOPD: 1.44 UPDRS III score: EOPD: 15.38 LOPD: 21.63	35013369

**Table 3:** Main findings of clinical studies.

**No**	**Author**	**Main results**
1	Mihara	• In peripheral lymphocytes: the percentage of NK cells (CD3-CD56+) of the PD group was higher • NK activity was not significantly different between the PD and HC groups; • The correlation between NK activity of PD patients and disease duration was positive; • The percentage of NKG2A+ cells among CD3-CD56+ NK cells in the PD group was statistically lower; • No significant difference between both groups in the percentage of NKG2D+ cells amongCD3-CD56+ NK cells
2	Niwa	• In peripheral lymphocytes: the percentage of CD16+ and CD56+ cells was significantly higher in patients with PD • No significant difference between both groups in the NK cell activity; • The percentages of CD16+ and CD56+ cells were not correlated with the UPDRS and UPDRS III score;
3	Stevens	In PBMC: no changes in the numbers of CD56+ cells in PD patients
4	Cen	In PBMC: • The percentage of NK cells in peripheral blood from PD patients was higher than that from the control group; • The percentage of NK cells did not differ between the early-onset groups and the late-onset group
5	Sun	In peripheral blood lymphocytes: • The percentage of NK cells (CD3-CD16+CD56+) in PD patients was significantly higher than that in healthy controls; • People with NK cells deviating from the reference range had an increased risk of PD
6	Anderson, K. M.	• High expression KIR3DL1 alleles in combination with HLA-Bw4/Bw4i reduce gait difficulties and rigidity in Parkinson's disease; • Weak KIR3DL1/HLA interactions associate with higher risk of rigidity and lower risk of resting tremor; • High-expressing KIR3DL1*002 is at higher frequency in patients who present with symptoms related to movement
7	Huang	• The fraction of resting NK cells in PD was significantly higher • NK cell mediated cytotoxic pathways were enriched in PD; • The mRNA level of prostaglandin D2 synthase (PTGDS) was positively correlated with the fraction of resting NK cells
8	Konstantin Nissen	An increase in CD16 median fluorescence intensity (MFI) on the mature NK cells(CD3-CD56^dim^CD16+)during early PD (within 5 years of diagnosis);
9	Tian	In PBMC: • C32 (CD56+CD16+CD57-CD28-) cluster and C27 (CD56+CD16+CD57+CD28-) cluster increased in PD patients compared to those in HCs and both increased in patients with LOPD compared to those in EHCs; • C29 (CD56+CD16+CD57+CD28+) cluster was lower in patients with PD with no significant difference • Increase in C32 NK cells was associated with increased UPDRS and UPDRS-III scores, but decreased MMSE scores of PD patients;

### Number of NK Cells in PD

Seven studies analyzed NK cell count in the peripheral blood. One of them found no differences between the patients and controls (Stevens et al., 2012), five of them reported a higher percentage of NK cells in the patients with PD (Mihara et al., 2008; Niwa et al., 2012; Cen et al., 2017; Sun et al., 2019; Huang et al., 2021), and in one study, Tian et al. divided NK cells into three clusters and reported that only C32 (CD56+, CD16+, CD57–, and CD28–) and C27 (CD56+, CD16+, CD57+, and CD28–) clusters increased (Tian et al., 2022). Furthermore, we noticed that the markers used to identify NK cells from the PBMC were quite different. CD16, a typical NK cell marker and also known as FCγRIII, is an activation receptor that mediates ADCC effects. CD56, known as nerve cell-associated adhesion molecule (NCAM), is considered as a functional indicator of human NK cells and separates the cells into precursor and mature populations based on its expression. In some studies, cells labeled by CD16 or CD56 were used as markers, while in others, those cells that expressed both CD16 and CD56 were recognized as NK cells. Mihara et al. and Niwa et al. reported the occurrence of the inhibitory NKG2A cells and the activating NKG2D cells, which indicated that the percentage of NKG2A+ cells among CD3-CD56+ NK cells was lower and the percentage of NKG2D+ cells in the peripheral lymphocytes was higher in the PD group (*p* < 0.05) (Mihara et al., 2008; Niwa et al., 2012).

### NKCC in PD

Experiments and bioinformatics analysis were used to analyze the cytotoxicity in NK cells. Only two studies measured NKCC activity against the K562 target cells through the LDH cytotoxic assay and the calcein acetoxymethyl ester release assay, and both of them found no significant differences between the patients with PD and the controls (Mihara et al., 2008; Niwa et al., 2012). Additionally, the former study provided information that NKCC increased as the disease progressed in PD (Mihara et al., 2008). On the other hand, from the perspective of bioinformatics analysis, Huang et al. carried out the Kyoto Encyclopedia of Genes and Genomes (KEGG) pathway enrichment analysis and revealed that NK cell-mediated cytotoxic pathways were enriched in PD based on the blood expression profile (Huang et al., 2021).

### Correlation of NK Cells With Clinical Phenotypes

Since aging is a common predisposing factor for Parkinson's disease, stratification by age at disease onset needs to be considered (Pagano et al., 2016). Two studies analyzed the subset of NK cells in the early-onset Parkinson's disease (EOPD) and late-onset Parkinson's disease (LOPD). Cen et al. observed that the percentage of NK cells in peripheral blood did not differ significantly between the two groups (*p* = 0.067) (Cen et al., 2017). Tian et al. showed a more detailed comparison between early- or late-onset patients and the age-matched controls. For the less toxic NK cells, CD32 (CD56+, CD16+, CD57–, and CD28–) clusters were significantly higher in the EOPD/LOPD group, while CD27 (CD56+, CD16+, CD57+, and CD28–) clusters were significantly higher only in the LOPD group. Cytotoxic CD29 (CD56+, CD16+, CD57+, and CD28+) clusters were lower in the LOPD group when compared to the control group, with no statistical difference between the groups. Furthermore, it was found that C27 cell clusters increased as the disease was prolonged in LOPD (Tian et al., 2022).

In addition, the disease severity may also be associated with the NK cells. Two studies explored the relationship between NK cells and disease progression measured by UDPRS and Mini-Mental State Examination (MMSE) scales. According to Niwa et al., the percentage of CD16+ cells and CD56+ cells was not correlated with the UPDRS (*p* > 0.1) and UPDRS-III scores (*p* > 0.1) in the 29 patients diagnosed with sporadic PD (Niwa et al., 2012). However, an increase in C32 clusters was found to correspond to increased UPDRS and UPDRS-III scores but decreased MMSE scores in the patients with PD (Tian et al., 2022).

In addition, other markers of NK cells were found to have an impact on the clinical manifestations. Using the bioinformatics tools, Anderson et al. analyzed the interaction between polymorphic killer immunoglobulin-like receptors (KIR) and human leukocyte antigen (HLA) class I ligands in patients with PD (Anderson et al., 2020). KIR3DL1, a member of the KIR family, conducts inhibitory signals depending on two immunoreceptor tyrosine-based inhibition motifs (ITIM) and binds to HLA-B molecules containing the Bw4 phenotype. The researchers found that high expression of KIR3DL1 alleles in combination with HLA-Bw4/Bw4i reduced gait difficulties and rigidity in patients, while weak KIR3DL1/HLA interactions were found to be associated with a higher risk of rigidity. Thus, they speculated that high expression levels of inhibitory KIR to reduce NK cell-mediated inflammation may reduce the severity of symptoms and have a protective effect in PD.

## Discussion

This is the first systematic review to evaluate the influence of NK cells in the development of Parkinson's disease based on the data from both foundation medicine and clinical medicine, and several issues should be considered critically and seriously.

### NK Cell Numbers and NKCC in PD

Evidence showed that NK cells were found in the brains of patients with PD (Earls et al., 2020), and the percentage of NK cells increased in the CNS parenchyma prior to the dopaminergic neuronal degeneration (Earls et al., 2019), which indicated infiltration of NK cells in the CNS during PD progression. From the point of ethics and experimental convenience, NK cells in the peripheral blood were appropriate and easy to obtain and could also give us information about pathogenesis. However, analysis of the cell counts and cell cytotoxicity had produced some contradictory results in both clinical studies and animal studies.

Most groups reported increased NK cells (Mihara et al., 2008; Niwa et al., 2012; Cen et al., 2017; Sun et al., 2019; Huang et al., 2021; Tian et al., 2022) in patients, while some researchers showed that the number of NK cells did not change significantly when compared to the corresponding controls (Stevens et al., 2012; Earls et al., 2019; Grembecka et al., 2021). We speculated that cohort/model differences, PD subtype, disease duration, or time delay between the clinical measurements of the disease and the pathological changes are the possible influencing factors, and the mechanisms underlying altered immune profiles still need to be explored.

Moreover, peripheral blood mononuclear cells (PBMCs) were shown as a basic reference in most studies. PBMCs are cell groups that include blood cells with a round nucleus (i.e., lymphocytes, monocytes, NK cells, or dendritic cells) (Sen et al., 2017). Among the PBMCs, components other than NK cells may also be altered in the pathological process of PD. For example, changes in monocyte subpopulations were observed in the early and late PD (Harms et al., 2021; Tian et al., 2022). Therefore, it might be better to measure the absolute value of the NK cells for comparison rather than considering only the percentage of cells.

As for the NKCC, Huang et al. confirmed enriched NK cell-mediated cytotoxic pathways in the peripheral blood using Gene Set Enrichment Analysis (GSEA), supporting a disease-associated cytotoxic response of NK cells in PD (Huang et al., 2021). Also, NK cells may be actively involved in the acute injury of dopaminergic neurons, since increased peripheral NKCC was found in rats injected with 6-OHDA for several weeks (Grembecka et al., 2021). However, some studies revealed no significant changes in NKCC targeting the K562 cells lacking ligands of the inhibitory NKG2A receptors (Mihara et al., 2008; Niwa et al., 2012). Given the fact that alterations in the expression levels of NKG2A receptors on NK cells were inconsistent between different studies (Mihara et al., 2008; Niwa et al., 2012), a possible approach involving the measurement of NKCC activity would be an influencing factor in the final results.

Notably, researchers for the first time observed that extracellular α-syn aggregates are internalized and degraded in the NK cells, and a more intriguing finding is that NKCC decreased obviously in a dose-dependent manner after the interaction between α-syn aggregates and NK cells (Earls et al., 2020). It seemed that the internalization of α-syn modulated the cytotoxicity of NK cells in a non-activated way. Recent studies have demonstrated that microglia can ingest and degrade extracellular α-syn with high efficiency, but consequently, α-syn fibrils are likely to induce apoptosis of microglia by increased reactive oxygen species (ROS) production and mitochondrial network disintegration (Choi et al., 2020; Scheiblich et al., 2021). In this case, whether α-syn exhibits toxicity in the NK cells and how α-syn aggregates affect the NKCC after the phagocytosis are still unclear.

In addition, researchers also observed increased CD16 expression on mature NK cells and speculated that an enhanced ADCC effect was primed in patients with PD (Konstantin Nissen et al., 2022). Since information about antibodies and target cells is unknown, cytotoxicity through ADCC is not measured in an intuitive way, and hence more number of experiments are needed to prove this hypothesis. Therefore, some doubts remain about the measurement of NKCC. To further elucidate these findings, mechanisms and further validations are encouraged to identify the cytotoxicity of NK cells more accurately and comprehensively in the dynamic development of PD in patients.

### NK Cells and Age at Onset in PD

Previous studies demonstrated that older age at onset was associated with a more severe motor phenotype and higher H&Y stage and UPDRS-III score, and similar results were observed by Cen et al. and Tian et el. (Szewczyk-Krolikowski et al., 2014; Pagano et al., 2016). Possibly, it can be explained by a greater dopaminergic dysfunction in patients with older age at onset, but the mechanisms are still unclear. Evidence suggests that genetic variation plays an important role in determining the age of onset of PD (Nalls et al., 2015). GWAS has implicated that SNCA, TMEM175, and GBA are associated with PD age at onset and are also implicated in α-syn aggregation pathways (Blauwendraat et al., 2019), which means that accumulation and clearance of α-syn are likely to be involved in the pathogenesis of age at onset. Besides, differences in the immune status exist in the patients with early- and late-onset PD (Tian et al., 2022). As for NK cells, functions alter with the differential expression of molecules on the cell surface in the relevant development stages and can be affected by various immune responses (Yu et al., 2013; Abel et al., 2018). It is possible that NK cells are related to age at onset in patients with PD.

Tian et al. analyzed peripheral mature NK cells marked by CD16 and CD56, and further distinguished cell maturity by expression of CD57 (a marker of terminal maturation) and CD28 (critical for co-stimulation of the cells to proliferate and produce IFN-γ) (Tian et al., 2022). Cytotoxicity of cells marked by CD57+CD28+ (CD29 clusters) is higher than those marked by CD57+CD28- (CD27 clusters) and CD57-CD28- (CD32 clusters). Interestingly, the researchers found higher CD27 and CD32 cell clusters and lower CD29 clusters in LOPD, while only higher CD32 clusters were noticed in EOPD, which suggested that age at onset might be associated with the development and toxicity alterations of NK cells in PD. Furthermore, considering that aging is one of the main risk factors for PD (Pagano et al., 2016; Poewe et al., 2017), it is crucial to control the variables such as age-matched healthy controls in the study design involving comparison between EOPD and LOPD.

### NK Cells and Clinical Symptoms in PD

NK cells are essential effectors in innate immunity, since NK cells can infiltrate the CNS during PD progression (Earls et al., 2020). However, whether there are pathophysiological reflections on the clinical motor and non-motor symptoms is an important issue for us to understand the role of NK cells in PD. Tian et al. observed less toxic NK cells increased with more severe motor and cognitive dysfunctions based on the evaluation of UPDRS-III and MMSE scales (Tian et al., 2022). Anderson et al. found that high expression of inhibitory KIR on NK cells might protect against severe motor symptoms in patients (Anderson et al., 2020). Additionally, in the PFF α-syn model, increased motor symptoms, exacerbated motor deficits, and increased p-α-syn inclusions were observed due to NK cell depletion (Earls et al., 2020). Therefore, we speculated that NK cells carry out killing and phagocytosis in a less toxic or non-hyperactivated way, which tend to play a protective role in the progression of PD.

However, previous studies have demonstrated that NK cells likely act as a double-edged sword in central nervous system disorders (Poli et al., 2013, 2018). On the one hand, they can induce the activation of neural cell death (Garofalo et al., 2020), while on the other hand, they have the ability to suppress CNS inflammation (Hao et al., 2010). In addition, immune dysfunction is quite complex in the development of PD. Pro-inflammatory or anti-inflammatory cytokines and cells, such as microglia, monocytes, T cells, B cells, and so on, are undoubtedly important participants in the pathological process and correlate with disease severity and disability in patients with PD (reviewed in detail in: Qin et al., 2016; Chen et al., 2018; Sabatino et al., 2019; Harms et al., 2021). However, only a few studies have explored their interactions with NK cells in PD till now, so the detrimental effect of NK cells is still uncertain and needs further investigation.

### Confounding Factors Associated With the Function of NK Cells in PD

Drugs are the influential factors in the study design. Basu et al. reviewed that dopamine and its receptor agonists altered the function of NK cells, for example, administration of dopamine increased the killing ability of specific NK cells (Basu and Dasgupta, 2000). Thus, data collection may be better in newly diagnosed PD patients to reduce the effects of dopamine replacement therapies. Additionally, neurotransmitters and neuroendocrine factors, such as glucocorticoids, serotonin, and epinephrine, have been reported to affect the activities of NK cells through a complex communication between the immune system and the nervous system (Capellino et al., 2020).

## Limitations

While we included the available studies on the relevant topic and systematically summarized the main results, there are some limitations in the article. First, heterogeneity of the inclusion criteria and experimental methods hindered a meta-analysis of the data, so we chose to report a descriptive analysis of our findings, rather than the quantitative conclusions. Second, a small sample size was found in most of the included studies, which reduced the reliability of the corresponding results. Third, the quality of some included studies was affected by several factors, such as lack of information on the basic characteristics of the patients, the absolute count of the measurement, and so on. Furthermore, publication bias toward studies with positive clinical outcomes cannot be ruled out.

## Future Directions and Conclusion

In the future, we hope that more promising translational therapeutic targets for PD will emerge with the conducting of extensive research on the essential pathological and physiological changes. It is known that NK cells can launch a rapid attack the next time they encounter the same antigen through antigen-mediated immune memory, and they can then present strong killing functions when perceiving a similar inflammatory environment. In addition, studies have shown that CNS-derived antigens can trigger neuroinflammation responses in PD. Therefore, there is a possibility that memory NK cells exist in the PD progression, and it is quite interesting to be explored in the future. In addition, NK cells have been reported to be associated with cognitive decline due to some complex mechanisms. Since non-motor symptoms, such as cognitive deficits, also have a certain impact on the life quality of patients with PD, looking for connections between NK cells and the non-motor symptoms is worthwhile in the basic experiments and clinical practice.

Overall, we reviewed the pathologic role of NK cells in Parkinson's disease systematically and summarized the characteristics of NK cell numbers, NKCC, and other surface molecules on the NK cells in patients with PD and animal models. Further, we analyzed the relationship between NK cells and the clinical manifestations in PD and speculated a probable neuroprotective role of NK cells. We hope that we have provided the necessary impetus for continued studies on the functions of NK cells in PD, and more potential biomarkers or targets will spring up during the diagnosis and treatment of PD in the future.

## Data Availability Statement

The raw data supporting the conclusions of this article will be made available by the authors, without undue reservation.

## Author Contributions

LZ, YZ, and DF: conceptualization. LZ and YZ: methodology and data curation. LZ: writing—original draft preparation. YZ and DF: writing—review and editing. All authors have read and agreed to the published version of the manuscript.

## Conflict of Interest

The authors declare that the research was conducted in the absence of any commercial or financial relationships that could be construed as a potential conflict of interest.

## Publisher's Note

All claims expressed in this article are solely those of the authors and do not necessarily represent those of their affiliated organizations, or those of the publisher, the editors and the reviewers. Any product that may be evaluated in this article, or claim that may be made by its manufacturer, is not guaranteed or endorsed by the publisher.

## References

[B1] Abel A. M. Yang C. Thakar M. S. Malarkannan S. (2018). Natural killer cells: development, maturation, and clinical utilization. Front. Immunol. 9, 1869. 10.3389/fimmu.2018.01869 30150991 PMC6099181

[B2] Anderson K. M. Augusto D. G. Dandekar R. Shams H. Zhao C. Yusufali T. . (2020). Killer cell immunoglobulin-like receptor variants are associated with protection from symptoms associated with more severe course in parkinson disease. J. Immunol. 205, 1323–1330. 10.4049/jimmunol.2000144 32709660 PMC7484130

[B3] Basu S. Dasgupta P. S. (2000). Dopamine, a neurotransmitter, influences the immune system. J. Neuroimmunol. 102, 113–124. 10.1016/S0165-5728(99)00176-9 10636479

[B4] Blauwendraat C. Heilbron K. Vallerga C. L. Bandres-Ciga S. Von Coelln R. Pihlstrøm L. . (2019). Parkinson's disease age at onset genome-wide association study: Defining heritability, genetic loci, and α-synuclein mechanisms. Mov. Disord. 34, 866–875. 10.1002/mds.27659 30957308 PMC6579628

[B5] Bokor M. Farag,ó A. Garam T. Malatinszky G. Schnabel R. (1993). Antibody-dependent cell-mediated cytotoxicity (ADCC) in Parkinson's disease. J. Neurol. Sci. 115, 47–50. 10.1016/0022-510X(93)90065-7 8468591

[B6] Brakedal B. Tzoulis C. Haugarvoll K. (2021). A nationwide study of the incidence, prevalence and mortality of Parkinson's disease in the Norwegian population. Eur. J. Neurol. 28, 81–81. 10.1038/s41531-022-00280-4 35236852 PMC8891365

[B7] Capellino S. Claus M. Watzl C. (2020). Regulation of natural killer cell activity by glucocorticoids, serotonin, dopamine, and epinephrine. Cell Mol. Immunol. 17, 705–711. 10.1038/s41423-020-0477-9 32503998 PMC7331581

[B8] Cen L. Yang C. Huang S. Zhou M. Tang X. Li K. . (2017). Peripheral lymphocyte subsets as a marker of Parkinson's disease in a Chinese population. Neurosci. Bull. 33, 493–500. 10.1007/s12264-017-0163-9 28791571 PMC5636734

[B9] Chen Z. Chen S. Liu J. (2018). The role of T cells in the pathogenesis of Parkinson's disease. Prog. Neurobiol. 169, 1–23. 10.1016/j.pneurobio.2018.08.002 30114440

[B10] Choi I. Zhang Y. Seegobin S. P. Pruvost M. Wang Q. Purtell K. . (2020). Microglia clear neuron-released α-synuclein via selective autophagy and prevent neurodegeneration. Nat. Commun. 11, 1386. 10.1038/s41467-020-15119-w 32170061 PMC7069981

[B11] Earls R. H. Menees K. B. Chung J. Barber J. Gutekunst C. A. Hazim M. G. . (2019). Intrastriatal injection of preformed alpha-synuclein fibrils alters central and peripheral immune cell profiles in non-transgenic mice. J. Neuroinflammation 16, 250. 10.1186/s12974-019-1636-8 31796095 PMC6889316

[B12] Earls R. H. Menees K. B. Chung J. Gutekunst C. A. Lee H. J. Hazim M. G. . (2020). NK cells clear alpha-synuclein and the depletion of NK cells exacerbates synuclein pathology in a mouse model of alpha-synucleinopathy. Proc. Natl. Acad. Sci. U. S. A. 117, 1762–1771. 10.1073/pnas.1909110117 31900358 PMC6983411

[B13] Feigin V. L. Vos T. Alahdab F. Amit A. M. L. Bärnighausen T. W. Beghi E. . (2021). Burden of neurological disorders across the US From 1990-2017: a global burden of disease study. JAMA Neurol. 78, 165–176. 10.1001/jamaneurol.2020.4152 33136137 PMC7607495

[B14] Garofalo S. Cocozza G. Porzia A. Inghilleri M. Raspa M. Scavizzi F. . (2020). Natural killer cells modulate motor neuron-immune cell cross talk in models of amyotrophic lateral sclerosis. Nat. Commun. 11, 1773. 10.1038/s41467-020-15644-8 32286313 PMC7156729

[B15] Grembecka B. Glac W. Listowska M. Jerzemowska G. O. D. Plucinska K. Majkutewicz I. . (2021). Subthalamic deep brain stimulation affects plasma corticosterone concentration and peripheral immunity changes in rat model of Parkinson's disease. J. Neuroimmune Pharmacol. 16, 454–469. 10.1007/s11481-020-09934-7 32648088 PMC8087570

[B16] Gruden M. A. Sewell R. D. E. Yanamandra K. Davidova T. V. Kucheryanu V. G. Bocharov E. V. . (2011). Immunoprotection against toxic biomarkers is retained during Parkinson's disease progression. J. Neuroimmunol. 233, 221–227. 10.1016/j.jneuroim.2010.12.001 21239064

[B17] Hao J. Liu R. Piao W. Zhou Q. Vollmer T. L. Campagnolo D. I. . (2010). Central nervous system (CNS)-resident natural killer cells suppress Th17 responses and CNS autoimmune pathology. J. Exp. Med. 207, 1907–1921. 10.1084/jem.20092749 20696699 PMC2931174

[B18] Harms A. S. Ferreira S. A. Romero-Ramos M. (2021). Periphery and brain, innate and adaptive immunity in Parkinson's disease. Acta Neuropathol. 141, 527–545. 10.1007/s00401-021-02268-5 33555429 PMC7952334

[B19] Huang Y. Liu H. Hu J. Han C. Zhong Z. Luo W. . (2021). Significant difference of immune cell fractions and their correlations with differential expression genes in Parkinson's disease. Front. Aging Neurosci. 13:686066. 10.3389/fnagi.2021.686066 34483877 PMC8416258

[B20] Iba M. Kim C. Sallin M. Kwon S. Verma A. Overk C. . (2020). Neuroinflammation is associated with infiltration of T cells in Lewy body disease and α-synuclein transgenic models. J. Neuroinflammation 17, 214. 10.1186/s12974-020-01888-0 32680537 PMC7368752

[B21] Konstantin Nissen S. Farmen K. Carstensen M. Schulte C. Goldeck D. Brockmann K. . (2022). Changes in CD163+, CD11b+, and CCR2+ peripheral monocytes relate to Parkinson's disease and cognition. Brain Behav. Immun. 101, 182–193. 10.1016/j.bbi.2022.01.005 35026420

[B22] Mihara T. Nakashima M. Kurolwa A. Akitake Y. Ono K. Hosokawa M. . (2008). Natural killer cells of Parkinson's disease patients are set up for activation: A possible role for innate immunity in the pathogenesis of this disease. Parkinsonism Relat. Disord. 14, 46–51. 10.1016/j.parkreldis.2007.05.013 17702627

[B23] Nalls M. A. Escott-Price V. Williams N. M. Lubbe S. Keller M. F. Morris H. R. . (2015). Genetic risk and age in Parkinson's disease: continuum not stratum. Mov. Disord. 30, 850–854. 10.1002/mds.26192 25778492 PMC5217457

[B24] Niwa F. Kuriyama N. Nakagawa M. Imanishi J. (2012). Effects of peripheral lymphocyte subpopulations and the clinical correlation with Parkinson's disease. Geriatr. Gerontol. Int. 12, 102–107. 10.1111/j.1447-0594.2011.00740.x 21929737

[B25] Pagano G. Ferrara N. Brooks D. J. Pavese N. (2016). Age at onset and Parkinson disease phenotype. Neurology 86, 1400–1407. 10.1212/WNL.0000000000002461 26865518 PMC4831034

[B26] Poewe W. Seppi K. Tanner C. M. Halliday G. M. Brundin P. Volkmann J. . (2017). Parkinson disease. Nat. Rev. Dis. Primers 3, 17013. 10.1038/nrdp.2017.13 28332488

[B27] Poli A. Kmiecik J. Domingues O. Hentges F. Blery M. Chekenya M. . (2013). NK cells in central nervous system disorders. J. Immunol. 190, 5355–5362. 10.4049/jimmunol.1203401 23687193

[B28] Poli A. Michel T. Patil N. Zimmer J. (2018). Revisiting the Functional Impact of NK Cells. Trends Immunol. 39, 460–472. 10.1016/j.it.2018.01.011 29496432

[B29] Prager I. Watzl C. (2019). Mechanisms of natural killer cell-mediated cellular cytotoxicity. J. Leukoc. Biol. 105, 1319–1329. 10.1002/JLB.MR0718-269R 31107565

[B30] Qin X. Y. Zhang S. P. Cao C. Loh Y. P. Cheng Y. (2016). Aberrations in peripheral inflammatory cytokine levels in Parkinson disease: a systematic review and meta-analysis. JAMA Neurol. 73, 1316–1324. 10.1001/jamaneurol.2016.2742 27668667

[B31] Sabatino J. J. Jr Probstel A. K. Zamvil S. S. (2019). B cells in autoimmune and neurodegenerative central nervous system diseases. Nat. Rev. Neurosci. 20, 728–745. 10.1038/s41583-019-0233-2 31712781

[B32] Schapira A. H. V. Chaudhuri K. R. Jenner P. (2017). Non-motor features of Parkinson disease. Nat. Rev. Neurosci. 18, 509. 10.1038/nrn.2017.62 28720825

[B33] Scheiblich H. Dansokho C. Mercan D. Schmidt S. V. Bousset L. Wischhof L. . (2021). Microglia jointly degrade fibrillar alpha-synuclein cargo by distribution through tunneling nanotubes. Cell 184, 5089–5106.e21. 10.1016/j.cell.2021.09.007 34555357 PMC8527836

[B34] Sen P. Kemppainen E. Oreši,č M. (2017). Perspectives on systems modeling of human peripheral blood mononuclear cells. Front. Mol. Biosci. 4, 96. 10.3389/fmolb.2017.00096 29376056 PMC5767226

[B35] Stevens C. H. Rowe D. Morel-Kopp M. C. Orr C. Russell T. Ranola M. . (2012). Reduced T helper and B lymphocytes in Parkinson's disease. J. Neuroimmunol. 252, 95–99. 10.1016/j.jneuroim.2012.07.015 22910543

[B36] Sun C. Zhao Z. Yu W. Mo M. Song C. Si Y. . (2019). Abnormal subpopulations of peripheral blood lymphocytes are involved in Parkinson's disease. Ann. Transl. Med. 7, 637. 10.21037/atm.2019.10.105 31930038 PMC6944630

[B37] Szewczyk-Krolikowski K. Tomlinson P. Nithi K. Wade-Martins R. Talbot K. Ben-Shlomo Y. . (2014). The influence of age and gender on motor and non-motor features of early Parkinson's disease: initial findings from the Oxford Parkinson Disease Center (OPDC) discovery cohort. Parkinsonism Relat. Disord. 20, 99–105. 10.1016/j.parkreldis.2013.09.025 24183678

[B38] Tan E. K. Chao Y. X. West A. Chan L. L. Poewe W. Jankovic J. (2020). Parkinson disease and the immune system - associations, mechanisms and therapeutics. Nat. Rev. Neurol. 16, 303–318. 10.1038/s41582-020-0344-4 32332985

[B39] Tian J. Dai S. B. Jiang S. S. Yang W. Y. Yan Y. Q. Lin Z. H. . (2022). Specific immune status in Parkinson's disease at different ages of onset. NPJ Parkinsons Dis. 8, 5. 10.1038/s41531-021-00271-x 35013369 PMC8748464

[B40] Vekrellis K. Xilouri M. Emmanouilidou E. Rideout H. J. Stefanis L. (2011). Pathological roles of alpha-synuclein in neurological disorders. Lancet Neurol. 10, 1015–1025. 10.1016/S1474-4422(11)70213-7 22014436

[B41] Wong Y. C. Krainc D. (2017). alpha-synuclein toxicity in neurodegeneration: mechanism and therapeutic strategies. Nat. Med. 23, 1–13. 10.1038/nm.4269 28170377 PMC8480197

[B42] Yu J. Freud A. G. Caligiuri M. A. (2013). Location and cellular stages of natural killer cell development. Trends Immunol. 34, 573–582. 10.1016/j.it.2013.07.005 24055329 PMC3852183

[B43] Zitti B. Bryceson Y. T. (2018). Natural killer cells in inflammation and autoimmunity. Cytokine Growth Factor Rev. 42, 37–46. 10.1016/j.cytogfr.2018.08.001 30122459

