# Supplementary material for: Pathological Role of Natural Killer Cells in Parkinson's Disease: A Systematic Review
Source: Front Aging Neurosci. 2022 May 18;14:890816. doi: 10.3389/fnagi.2022.890816 (PMC9157643; doi:10.3389/fnagi.2022.890816)
Supplement: Supplementary file 1 [file Table_1.docx]

**Supplementary materials**

**eTable 1 Search strategy of the databases**

| Database | Search strategy |
| --- | --- |
| PubMed | #1 Parkinson disease [MeSH Terms]  #2 Parkinson disease [Title/Abstract] OR Parkinson’s disease [Title/Abstract]  #3 alpha-Synuclein [Mesh Terms]  #4 alpha-Synuclein [Title/Abstract] OR alpha-synucleinopathy [Title/Abstract]  #5 dopaminergic nerve [Title/Abstract]  #6 #1 OR #2 OR #3 OR #4 OR #5  #7 Killer Cells, Natural [MeSH Terms]  #8 Natural killer cells [Title/Abstract] OR Natural killer cell [Title/Abstract] OR NK cells [Title/Abstract] OR NK cell [Title/Abstract]  #9 #7 OR #8  #10 #6 AND #9 |
| Embase | #1 ‘parkinson disease’/exp OR ‘parkinson disease’  #2 ‘alpha-synuclein’  #3 ‘synucleinopathy’  #4 ‘dopaminergic nerve cell’  #5 #1 OR #2 OR #3 OR #4  #6 ‘natural killer cell’  #7 #5 AND #6 |
| Web of Science | #1 TS=(Parkinson disease) OR TS=(Parkinson’s disease)  #2 TS=(dopaminergic nerve )  #3 TS=( alpha-synuclein) OR TS=(alpha-synucleinopathy)  #4 #1 OR #2 OR #3  #5 TS=(Killer Cells, Natural) OR TS=(Natural killer cells) OR TS=(NK cell) OR TS=(Natural killer cell)  #6 #4AND #5 |

**eTable 2 Quality assessment for animal studies** **(CAMARADES)**

| No. | Judgement | ID1 | ID2 | ID3 |
| --- | --- | --- | --- | --- |
| 1 | sample size calculation | 0 | 0 | 0 |
| 2 | random allocation to treatment or control | 0 | 0 | 0 |
| 3 | allocation concealment | 0 | 0 | 0 |
| 4 | blinded assessment of outcome | 1 | 1 | 0 |
| 5 | appropriate animal model | 1 | 1 | 1 |
| 6 | use of anesthetic without significant intrinsic neuroprotective activity | 1 | 1 | 1 |
| 7 | statement of control of temperature | 1 | 1 | 1 |
| 8 | compliance with animal welfare regulations | 1 | 1 | 1 |
| 9 | peer-reviewed publication | 1 | 1 | 1 |
| 10 | statement of potential conflict of interests | 1 | 1 | 1 |
| Score |  | 7 | 7 | 6 |

**eTable 3 Quality assessment for clinical studies (NOS)**

| Item | No. | ID1 | ID2 | ID3 | ID4 | ID5 | ID6 | ID7 | ID8 | ID9 |
| --- | --- | --- | --- | --- | --- | --- | --- | --- | --- | --- |
| Selection | 1 | 1 | 1 | 1 | 1 | 1 | 1 | 1 | 1 | 1 |
|  | 2 | 1 | 0 | 0 | 0 | 0 | 0 | 0 | 0 | 0 |
|  | 3 | 1 | 0 | 0 | 0 | 0 | 0 | 1 | 1 | 0 |
|  | 4 | 1 | 1 | 1 | 1 | 1 | 1 | 1 | 1 | 1 |
| Comparability | 5 | 2 | 2 | 2 | 2 | 2 | 1 | 1 | 1 | 2 |
| Exposure | 6 | 1 | 1 | 1 | 1 | 1 | 1 | 1 | 1 | 1 |
|  | 7 | 1 | 1 | 1 | 1 | 1 | 1 | 1 | 1 | 1 |
|  | 8 | 1 | 1 | 1 | 1 | 1 | 1 | 1 | 0 | 1 |
| Score | - | 9 | 7 | 7 | 7 | 7 | 6 | 7 | 6 | 7 |
